# Dynamic Characteristics of a Hydraulic Amplification Mechanism for Large Displacement Actuators Systems

**DOI:** 10.3390/s100402946

**Published:** 2010-03-29

**Authors:** Xavier Arouette, Yasuaki Matsumoto, Takeshi Ninomiya, Yoshiyuki Okayama, Norihisa Miki

**Affiliations:** School of Integrated Design Engineering, Keio University, 3-14-1 Hiyoshi, Kohoku-ku, Yokohama, Kanagawa, 223-8522, Japan; E-Mails: xavi@miki.mech.keio.ac.jp (X.A.); matsumoto@miki.mech.keio.ac.jp (Y.M); ninomiya@miki.mech.keio.ac.jp (T.N.); okayama@miki.mech.keio.ac.jp (Y.O.)

**Keywords:** hydraulic amplification, liquid encapsulation, dynamic actuation, tactile display, MEMS

## Abstract

We have developed a hydraulic displacement amplification mechanism (HDAM) and studied its dynamic response when combined with a piezoelectric actuator. The HDAM consists of an incompressible fluid sealed in a microcavity by two largely deformable polydimethylsiloxane (PDMS) membranes. The geometry with input and output surfaces having different cross-sectional areas creates amplification. By combining the HDAM with micro-actuators, we can amplify the input displacement generated by the actuators, which is useful for applications requiring large deformation, such as tactile displays. We achieved a mechanism offering up to 18-fold displacement amplification for static actuation and 12-fold for 55 Hz dynamic actuation.

## 1. Introduction

Microelectromechanical systems (MEMS) technology has proven its abilities for providing miniaturized, integrated, fast responding and energy-saving devices. These qualities can be combined to develop tactile displays, an emerging human interface application, which artificially produces tactile stimuli [1–7]. Tactile displays are being developed to offer a new human-machine interface approach for virtual reality applications and interaction devices such as mice or games controllers but also to supporting visually impaired persons. They consist of micro-actuator arrays that mechanically stimulate skin receptors to deliver tactile information. To dynamically stimulate tactile receptors, a skin deformation over 100 μm is required, but low-power MEMS actuators offer only 10 μm displacement. So in order to create larger stimuli, we need a mechanism capable of increasing the actuator’s movements ten-fold. Also, because information transmission by tactile receptors is highly dependent on stimulus frequency we have to determine the factors influencing the dynamic characteristics of this large displacement actuator system.

High-flow-rate microvalves are another MEMS application for compact high-performance hydraulic pumping systems that requires large-displacement actuation. To achieve these microvalves, piezoelectrically driven hydraulic displacement-amplification mechanisms are generally used and one such mechanism reported for microvalves is a hydraulic amplification that utilizes incompressible fluid in a microchamber whose input surface is larger than its output surface [8–12].

In our previous work [13], we devised a mechanism using a totally encapsulated liquid able to amplify displacements up to 15-fold. It consisted of a microcavity with different cross-sectional areas, etched in a silicon wafer, and containing a liquid sealed by two easily deformable thin polydimethylsiloxane (PDMS) membranes. A displacement applied to the input surface was amplified at the smaller output surface.

**Figure 1.**
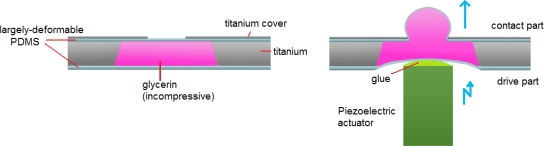
Schematic cross-sectional views of our HDAM.

In this article, we describe the characteristics of an optimized hydraulic displacement amplification mechanism (HDAM) consisting of a microchamber etched in a titanium wafer and filled with glycerin sealed by easily deformable thin PDMS membranes. On the top, a small opening etched in a titanium plate covers the chamber, as shown in the left part of Figure 1. A micro-actuator’s displacement applied to the large-area bottom of the microcavity (the drive part) is amplified at the small-area of the top titanium plate (the contact part) (right part of Figure 1). Compared with previous work using brittle silicon layers to form microcavities, the device created here is only made of titanium and PDMS and thus, simpler and less fragile. In order to obtain the greatest displacement amplification performance, we tested devices with various top PDMS membrane thicknesses and contact part sizes. To explain the results, we investigated the influence of the components’ dimensions on membrane deformation using the Finite Element Method (FEM). Once optimization was done, to characterize dynamically the HDAM, varying design parameters of top PDMS membrane thickness and chamber depth, we measured the consequences on amplification ratio (= amplified displacement/driving displacement), depending on actuator frequency. Then, by combining low-power actuators with the HDAM, the resulting displacement could fulfill tactile receptors’ requirements and recreate tactile sensations. Also for tactile displays applications, our HDAM being placed between skin and the micro-actuators, it offers the advantage of electrically and thermally insulating the actuators from the fingers.

## 2. Design and Fabrication

### 2.1. Design

**Figure 2.**
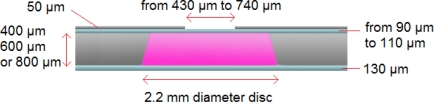
Dimensions of the HDAM.

When designing the HDAM, we chose to develop a mechanism usable for further tactile display applications. Then, considering the fact that we are only able to distinguish between two points when their mutual distance is larger than 3 mm, inter-actuator spacing was fixed as this two-point discrimination threshold. Consequently, we decided to design the contact part area lower than this distance as a 2.2 mm diameter disc. Also, the bottom PDMS membrane thickness was fixed to 130 μm because it has to be resistant enough to endure the direct contact and deformation created by actuators. The minimal titanium plate thickness not deformed during displacement amplification was found to be 50 μm. By doing so, we still have the possibility to modify and optimize three parameters to dynamically characterize the HDAM: diameter of the titanium plate opening, thickness of the top PDMS membrane and depth of the chamber (Figure 2). Titanium plate opening dimensions ranging from 430 μm to 740 μm diameter discs and top PDMS membrane thicknesses ranging from 90 μm to 110 μm have been tested in order to obtain the optimal amplification ratio. Three different depths of the chamber—400, 600 and 800 μm—have been obtained by bonding the appropriate number of 200 μm thick titanium wafers.

### 2.2. Fabrication Process

A 200-μm-thick titanium wafer was used to form the microcavity. The titanium wafer is etched by hydrofluoric acid (HF) using copper as a protective layer. However, noticing poor adherence between Ti and Cu in a HF environment, we use AZ photoresist as an adhesive layer to secure protection of the Ti by Cu during etching. We made a largely deformable PDMS polymer by mixing DC 3145 CLEAR and RTV thinner (Dow Corning Toray Inc.). DC 3145 CLEAR is a PDMS elastomer that can stretch more than 650%. The thinner was added in order to decrease the viscosity of the elastomer so that we could prepare appropriately thick membranes by spin coating. Good results were obtained when the ratio of the volumes of DC 3145 CLEAR and RTV thinner was 1:2. The results indicated that the Young's modulus of the largely-deformable PDMS was 32 kPa, while strains are small and it could stretch more than 450% [12]. This PDMS is much less stiff than the SILPOT 184 (Dow Corning Toray Inc.) often used in MEMS and was thought to be suitable for our HDAM. Given the large gas permeability of PDMS [14], glycerin presents the advantage over water of being non-volatile (vapor pressure of glycerol is 0.133 Pa at 25 °C compared to 3,173 Pa for water) so this ensures the long-term use of the mechanism.

**Figure 3.**
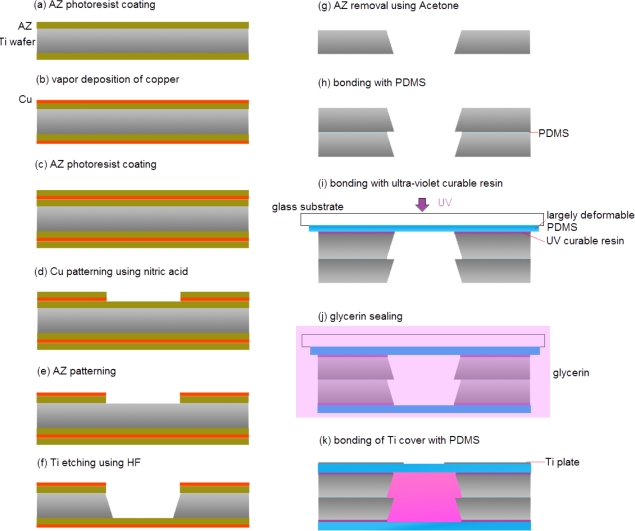
HDAM fabrication process.

The HDAM fabrication process is shown in Figure 3: (a) a Ti wafer was coated with OAP (Tokyo Ohka Kogyo Inc.) as a primer, a positive AZ was applied by spin coating. (b) Copper film is created on the top of AZ using vacuum vapor deposition. (c) Once again, AZ photoresist was coated on the copper layer. (d) The AZ photoresist was patterned by photolithography. The Cu layer was etched using nitric acid (HNO_3_) 1:20 H_2_O. (e) The photoresist was removed by UV exposure and development processes. (f) The Ti wafer was etched using HF 1:10 H_2_O. (g) The AZ was removed using acetone (copper was stripped with AZ removal). (h) The microcavity was formed by using a thin layer of PDMS to bond two or more Ti wafers. (i) A thin PDMS membrane was formed on a glass substrate, bonded to the contact-part Ti wafer with UV-curable resin, and then exfoliated from the glass substrate.

This is the critical step of the fabrication process. Because of its brittle nature, successful exfoliation yield of the silicon layer was poor. To insure high-yield fabrication, we chose a titanium layer which is more ductile than silicon. (j) Glycerin was encapsulated in the chamber when a UV-curable resin was used to bond a thin PDMS membrane to the driver-part Ti wafer in a glycerin solution. This bonding-in-solution approach ensures that the encapsulated glycerin is bubble-free. (k) Ti plate etched with a small opening was bonded to PDMS membrane using a thin layer of PDMS.

## 3. Optimization of the Amplification Ratio

The first step to obtain a highly efficient amplification mechanism is to determine the influence of the three unfixed parameters on the amplification ratio to optimize our HDAM. Nevertheless, amplification is due to a top area smaller than the bottom one so if we only modify the depth of the chamber it will not influence the displaced volume by actuators nor the amplification ratio. This means we have to focus our study on the diameter of the contact part and the top PDMS membrane thickness.

### 3.1. Experimental Results

To determine which design affords the best amplification ratio, we fabricated devices with various contact part sizes for each of the HDAM created with 90, 95, 100, 105 and 110 μm-thick top PDMS membranes. The amplified displacement was measured optically with a digital microscope when the drive part was pushed up by a pin whose displacement was controlled precisely by a Z-axis stage.

**Figure 4.**
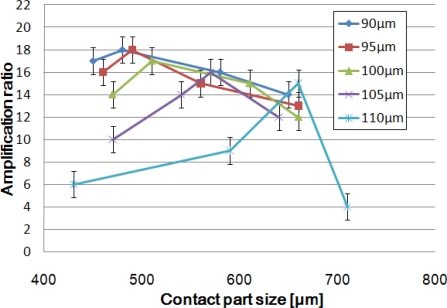
Amplification ratio depending on contact part size for different membrane thicknesses.

Figure 4 represents the amplification ratio depending on contact part size for different membrane thicknesses. The experiments show two results: first, we observe that the thinner the PDMS membrane is, the better the amplification; second, an optimal amplification ratio is obtained for one specific contact part size. Our best HDAM is able to create an up to 18-fold displacement amplification for a 90 μm-thick membrane and 460 μm-diameter contact part. Such a large displacement amplification is a great achievement in the field of micro-domain actuators.

A simple thinking about the mechanism used would lead us to think amplification ratio is linked to the cross-sectional ratio (drive part area over contact part area) and bigger cross-sectional ratio leads to better amplification. The experiments proved this to be wrong, so to understand the existence of an optimal design, we performed FEM analysis.

### 3.2. FEM Analysis

**Figure 5.**
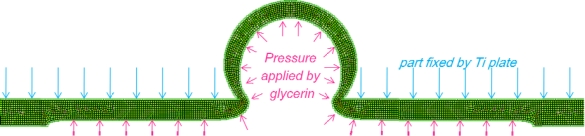
Deformed state of top PDMS membrane obtained by FEM simulation.

For FEM analysis, we used the MSC.MARC Mentat software to represent the deformed state of the top PDMS membrane under a uniform pressure for different membrane thicknesses and contact part sizes (Figure 5). PDMS membrane behavior was modeled by implementing a stress-strain curve obtained from a tensile test.

**Figure 6.**
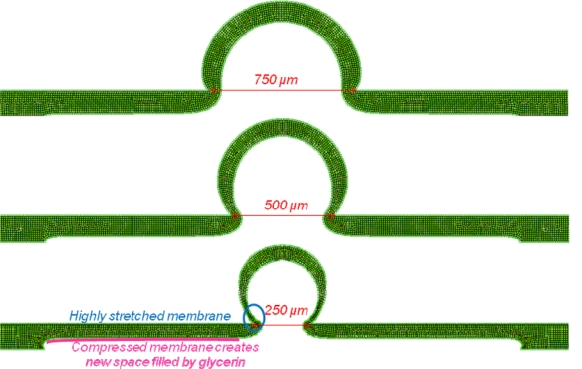
Deformed states obtained by FEM analysis for 750, 500 and 250 μm-contact part sizes with a 100 μm-thick PDMS membrane, same volume displacement.

For an identical volume of displaced liquid under the membrane, we can compare deformation shapes for different contact part sizes (Figure 6). According to the experimental results, we found an optimal value for contact part size. Explanation is found from captures of deformed membranes, too large contact area leads to small cross-sectional area ratio and too small contact area prevents the membrane from popping up. Indeed, when contact part area becomes too small, the emerged membrane part is extremely stretched and the membrane part located under the titanium plate is compressed by glycerin. Then, displaced liquid by PZT actuator remains under the compressed membrane and does not create greater amplification. Also, a thinner membrane limits compression effect so increases amplification ratio.

## 4. Dynamic Characteristics and Discussion

**Figure 7.**
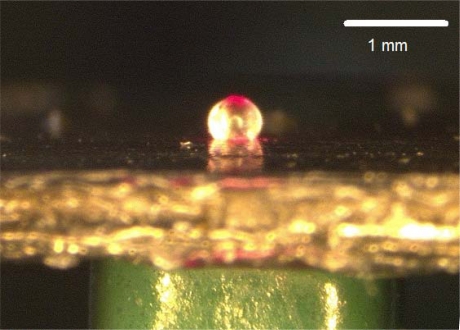
Deformation of membrane at the contact part by piezoelectric actuator under laser measurements.

The relation between the actuation frequency and the amplification ratio was investigated using HDAMs with different membrane thicknesses and HDAMs with various chamber depths. The driven displacement of the multilayer piezoelectric actuator (NEC-TOKIN Inc.) driven by a 100-V_p-p_ sine wave and the amplified displacement at the contact part were measured by a laser vibrometer, as shown in Figure 7.

**Figure 8.**
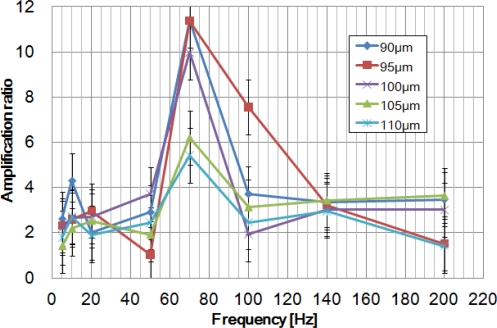
Amplification ratio depending on driving frequencies for different membrane thickness values.

The piezoelectric actuator was glued to the drive part of the HDAM to secure coordinate movements and prevent any bouncing effects between actuator and HDAM. To calculate the amplification ratio (= amplified displacement/driving displacement), after measuring the displacement profile of the PZT actuator without load depending on driving frequencies, we used these data to divide amplified displacement obtained by the HDAM coupled with the PZT actuator. Actually, the real amplification ratio is greater than the effective one we calculated because in our calculation, we used the driving displacement without load which is higher than the PZT actuator’s displacement loaded with the HDAM. Consequently, because we overvalued the driving displacement, we underestimated the amplification ratio and this is why we do not obtain an 18-fold amplification, even at a low frequency. Figure 8 shows amplification ratio depending on actuation frequency for the 90, 95, 100, 105 and 110 μm-thick membrane HDAMs with a 400 μm-depth chamber.

**Figure 9.**
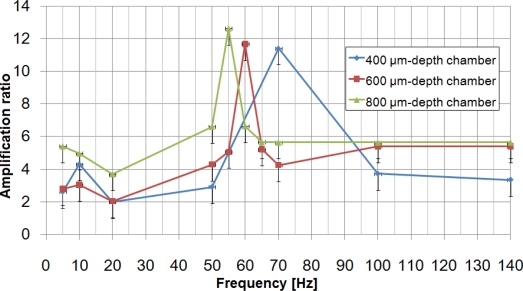
Amplification ratio depending on driving frequencies for three different chamber depths.

For each of them, resonance frequency was found to be 70 Hz, meaning that membrane thickness does not influence the resonance frequency of the HDAM. Excluding the resonance frequency, HDAM shows low performance for displacement amplification. Besides, at the resonance frequency, devices which showed better static amplification ratio (cf. Figure 6) also showed better dynamic amplification ratios. From this result, we can fix a 90 μm-thick membrane and 460 μm-diameter contact part as optimal parameters for the HDAM and focus on the study of the chamber’s geometry, the only parameter still not optimized. Using these parameters, three devices with 400, 600 and 800 μm-depth chamber have been created and tested (Figure 9). For each chamber depth, we performed measurements for three devices, and no significant difference in amplification ratio was observed from one HDAM to another: at resonance frequency, performance variation between devices can reach 1-point in amplification ratio; at other frequencies, variation is below this value. The best optimal amplification ratio is 12-fold for a 800 μm-depth chamber HDAM with 55 Hz driving frequency.

Using the simplest model where the HDAM is modeled as a spring-mass system where encapsulated fluid and membranes are respectively the mass *m* and the spring (spring constant *k*). Resonance frequency of the mechanism $\omega_{0}=\sqrt{k/m}$ varies as the inverse square root of the encapsulated liquid mass. When the chamber becomes deeper, the mass of the encapsulated liquid increases, therefore the resonance frequency decreases. Considering the resulting differences in encapsulated volume with 400, 600 and 800 μm-depth chambers, this explains the resonance frequency shift. Nevertheless, considering the viscoelastic behavior of the PDMS membrane at high rate deformation and the viscosity of glycerin, this simple model can only be used to predict shift of resonance frequency depending on liquid mass.

## 5. Conclusions

We have designed, fabricated, optimized and dynamically characterized a hydraulic displacement amplification mechanism (HDAM) that utilizes glycerin completely encapsulated by easily deformable PDMS. By conducting FEM analysis, we improved the performance of our HDAM achieving a 18-fold displacement amplification for static actuation. Once all the parameters influencing static amplification ratio were optimized, we dynamically characterized the HDAM, obtaining a 12-fold amplification ratio for a resonance frequency of 55 Hz. For tactile display applications, the displacement generated is enough for us to feel it. In our future work, we want to investigate sensations of human fingers.

## References

[1] Kwon H.J., Lee S.W., Lee S.S. Braille Code Display Device with a PDMS Membrane and Thermopneumatic Actuator.

[2] Yoshikawa W., Sasabe A., Sugano K., Tsuchiya T., Tabata O., Ishida A. Vertical Drive Micro Actuator Using SMA Thin Film for a Smart Button.

[3] Konyo M., Tadokoro S., Yoshida A., Saiwaki N. A Tactile Synthesis Method using Multiple Frequency Vibrations for Representing Virtual Touch.

[4] Kotani H., Takasaki M., Mizuno T. Surface Acoustic Wave Tactile Display Using a Large Size Glass Transducer.

[5] Biet M., Casiez G., Giraud F., Semail B. Discrimination of Virtual Square Gratings by Dynamic Touch on Friction Based Tactile Displays.

[6] Yokota H., Yamamoto A., Yamamoto H., Higuchi T. Producing Softness Sensation on an Electrostatic Texture Display for Rendering Diverse Tactile Feelings.

[7] Ohka M., Koga H., Mouri Y., Sugiura T., Miyaoka T., Mitsuya Y. (2007). Figure and Texture Presentation Capabilities of a Tactile Mouse Equipped with a Display Pad of Stimulus Pins. J. Adv. Mech. Des. Syst. Manuf.

[8] Roberts D.C., Hanqing L., Steyn J.L., Yaglioglu O., Spearing S.M., Schmidt M.A., Hagood N.W. (2003). A Piezoelectric Microvalve for Compact High-Frequency, High-Differential Pressure Hydraulic Micropumping Systems. J. Microelectromech. Syst.

[9] Kim H., Najafi K. Electrostatic Hydraulic Three-Way Gas Microvalve for High-Pressure Applications.

[10] Kim H., Najafi K. An Electrically-Driven, Large-Deflection, High-Force, Micro Piston Hydraulic Actuator Array for Large-Scale Microfluidic Systems.

[11] Wu X., Kim S.H., Ji C.H., Allen M.G. A Piezoelectrically-driven High Flow Rate Axial Polymer Microvalve with Solid Hydraulic Amplification.

[12] Kim H., Lee S., Najafi K. High-force Liquid-Gap Electrostatic Hydraulic Micro Actuators.

[13] Ninomiya T., Okayama Y., Matsumoto Y., Arouette X., Osawa K., Miki N. MEMS Tactile Display with Hydraulic Displacement Amplification Mechanism.

[14] Zhang Y., Ishida M., Kazoe Y., Sato Y., Miki N. (2009). Water Vapor Permeability Control of PDMS by the Dispersion of Collagen Powder. Trans. Electric. Electron. Eng.

